# Manual dexterity among older adults with and without sarcopenia

**DOI:** 10.1186/s12877-025-06862-0

**Published:** 2025-12-19

**Authors:** Shreyas Vignesh Rekha, Sidhiprada Mohapatra, Girish Nandakumar

**Affiliations:** 1 Department of Physiotherapy, Manipal College of Health Professions, Manipal Academy of Higher Education, Manipal, Karnataka India; 2 Co-cordinator, Center for Elder care, Education and Research on Elders, Manipal Academy of Higher Education, Manipal, India

**Keywords:** Aged, Long-term care settings, Muscle mass, Muscle strength, Purdue pegboard test

## Abstract

**Aim:**

The impact of sarcopenia on Manual Dexterity (MD) among older adults residing in long-term care settings (LTCS) is understudied. Hence, the objective is to compare MD using the Purdue Pegboard Test (PPT) among older adults with and without sarcopenia.

**Methods:**

A cross-sectional study was conducted in seven LTCS, and older adults were selected per the criteria decided a priori. Sarcopenia was evaluated through muscle mass, muscle strength, and physical performance using the Asian Working Group for Sarcopenia (AWGS) 2019 criteria, and MD was assessed using PPT.

**Results:**

Ninety-six older adults were included, with 83 identified as sarcopenic and 13 as non-sarcopenic. Sarcopenic participants exhibited significantly lower scores in PPT, ranging from 1.34 to 4.00 s across various sub-tasks, compared to non-sarcopenic participants.

**Conclusion:**

Sarcopenia significantly affects MD, with a decline of 12% to 33.9% in various dexterity tasks.

## Introduction

An emerging understanding of sarcopenia, once thought of as a geriatric condition, now considers it to be one of the non-communicable diseases (NCDs) [25]. With technological advancements, there is an increase in its detection rate, hence its rising incidence globally [32]. The pooled prevalence rate of sarcopenia among older adults ranges from 10 to 66 per cent, with variations as per the diagnostic criteria used [31]. A growing body of research on sarcopenia has identified various modifiable and non-modifiable risk factors [7]. It has been linked to numerous adverse health conditions, like osteoporosis [10], obesity [5], risk for falls [26], insulin resistance, and diabetes mellitus [14], CVDs [8], and CKD [27], affecting one’s Quality of Life (QoL) and increased mortality risk.

Manual dexterity (MD), the ability to make coordinated hand and finger movements to grasp and manipulate objects [21], is a significantly important function responsible for almost all functional activities like eating, dressing, grooming, writing, and self-hygiene [20]. The ability to move fingers independently and precisely, eye-hand coordination, bimanual control, finger isolation, in-hand manipulation, performing tasks without losing accuracy, and force modulation are a few of the many activities under manual dexterity [30]. Most of these MD activities are shown to be affected by aging and frailty [2].

The correlation between aging and reduced dexterity has been extensively studied in the literature [2]. The structural and functional age-associated changes in the neuromusculoskeletal system are most likely the cause of a decline in MD and have been reported in numerous clinical and scientific studies [23]. While frailty reflects a multidimensional deterioration involving physiological, metabolic, and functional domains [15], sarcopenia constitutes a distinct and quantifiable neuromuscular condition characterized by progressive reductions in muscle mass, strength, and performance [1]. Examining the relationship between sarcopenia and motor dexterity is therefore essential to delineate the specific neuromuscular mechanisms underlying fine motor impairment, independent of broader frailty processes. Despite its clinical relevance, the direct influence of sarcopenia on motor dexterity among older adults remains insufficiently explored. Hence, the objective of this study is to compare manual dexterity using the Purdue pegboard test (PPT) among older adults with and without sarcopenia.

## Materials and methods

This study followed a cross-sectional design conducted in Long-Term Care Settings (LTCS) in Udupi District, Karnataka State, India, from March 2023 to April 2024. Permissions from the authorities of LTCS were sought, and approval was obtained from the Institutional Ethics Committee, Kasturba Hospital, Manipal (IEC Number- 264/2023) and registered in the Clinical Trial Registry- India (CTRI/2023/09/057517).

### Participants

Older adults residing in LTCS, aged above 60 years, either sex, with a mini-COG score ≥ 3, a Barthel index ≥ 60 points, and those with functional vision were included. Residents with acute onset (less than 30 days) of cardiorespiratory disease, neurological disease, renal disease, critically or terminally ill, actively infected, having undergone recent hand surgery, or having deformities of the hands or knees or bed-bound or wheelchair bound were excluded from this study.

### Sample size

The sample size of *n* = 96 was estimated using the formula $\:n=\frac{[z1-\frac{\alpha\:}{2}+z1-\beta\:]}{\left(effect\:size\right)2}$, where 𝑧 (1−𝛼)/2 = 1.96; $\:1-\beta\:=0.84\:$and the effect size of 0.57 for the PPT.

### Assessment

After explaining the procedure, written informed consent was obtained from the participants. Demographic data, which included age, gender, height, weight, and hand dominance, were collected. Dexterity was evaluated using the PPT, which has four sub-tasks: performing with the dominant hand, non-dominant hand, both hands, and an assembly [19]. The participants were made to sit on a chair with a backrest, and the pegboard was placed on a table in front. A demonstration of the test and a practice trial were given. The participants were asked to retrieve a pin from a cup and place it into one of the holes on the board, beginning with the first hole that was closest to the cups. The objective was to fill as many holes as possible within a 30-second time limit, except for the assembly task, which is for 60 s. Three trials were given, and the number of pegs placed was noted, and the scores were averaged for each task.

Sarcopenia was assessed by AWGS (Asian Working Group for Sarcopenia) 2019 criteria: low muscle mass and either low physical performance and/or low muscle strength [3]. Skeletal muscle mass was estimated from Omron Karada Scan HBF-375 Bioelectrical Impedance Analyzer (BIA) measurements and expressed as skeletal muscle mass index (SMI) (SMI = skeletal muscle mass/body mass × 100). The AWGS-2019 cut-off values we used were as follows: <7.0 kg/m^2^ for male participants and < 5.7 kg/m^2^ for female participants. Muscle strength (grip) was assessed with a JAMAR digital handheld dynamometer (model no 2015090057) with the following cut-off values, proposed by AWGS-2019: <28.0 Kg for male participants and < 18.0 Kg for female participants. The grip strength of both hands was assessed following the standard criteria, and the average value was obtained. To evaluate physical performance, the five-time chair stand test (5-CST) was used. Participants were asked to perform sit-to-stand once, and if found to be comfortable, participants were requested to repeat it five times as quickly as possible, with the researcher recording the time taken to complete. Poor physical performance was considered as per the cut-off given by AWGS-2019, with participants taking ≥ 12 s [3].

### Statistical analysis

Data were analyzed using Jamovi (version 2.3.28). As the data did not follow a normal distribution, non-parametric statistical tests were employed. Descriptive statistics, including the median and interquartile range (IQR), were used to summarize demographic and clinical characteristics. Group comparisons were performed using the Mann–Whitney U test for continuous variables and the chi-square test of association for categorical variables. The Kruskal–Wallis test was applied to examine differences in manual dexterity among sarcopenic and non-sarcopenic older adults. The level of statistical significance was set at *p* ≤ 0.05.

## Results

**Fig. 1:**
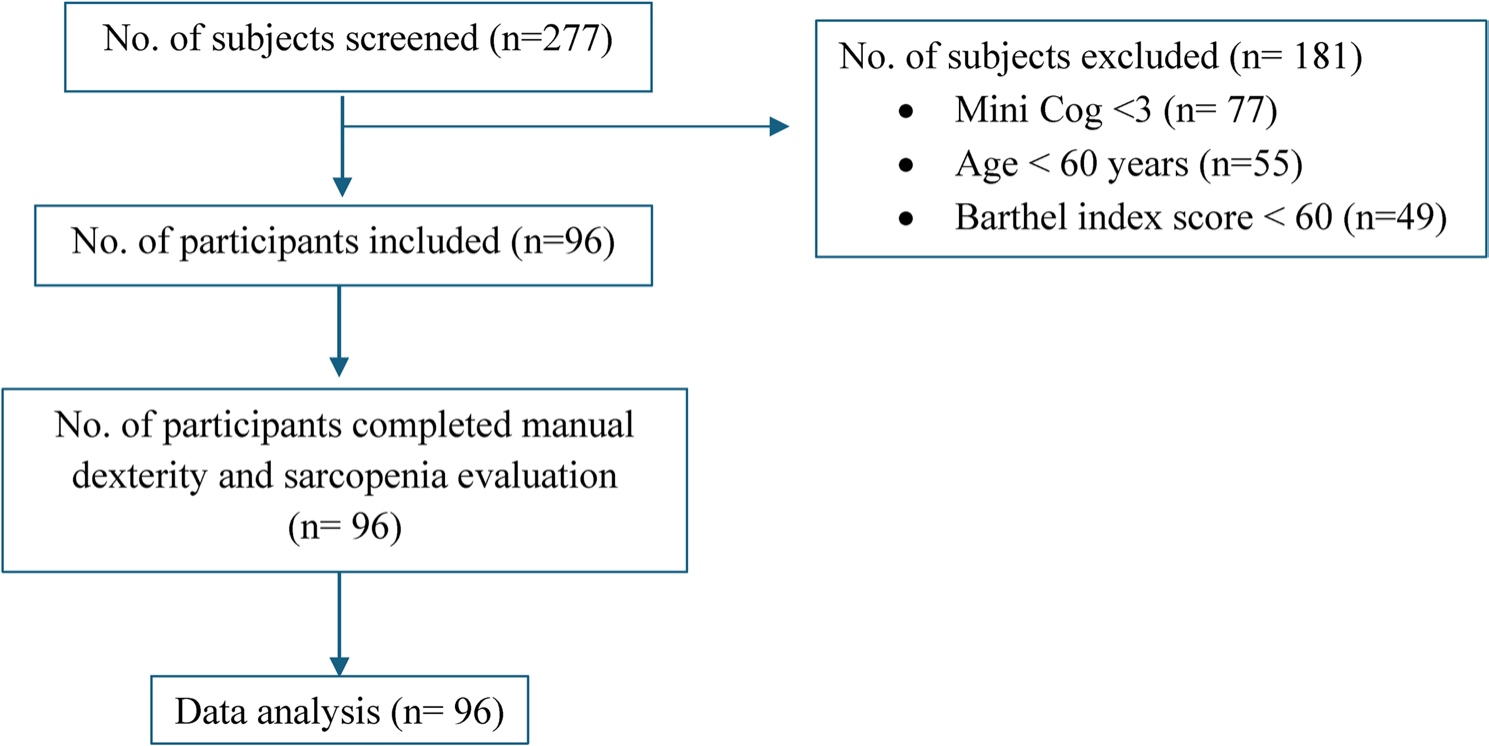
Flow of participants

Figure 1 shows the flow of participants. A total of *n* = 277 residents from eight LTCS of Udupi district, Karnataka, India were screened. Over 181 residents were excluded as they did not meet the eligibility criteria. A total of 96 participants were eligible to participate and were assessed in this study.

Table 1 represents the demographic characteristics of participants (*n* = 96) categorized as sarcopenics (*n* = 83) and non-sarcopenics (*n* = 13), more than 86% of the older adults residing in LTCS were sarcopenics. The older adults with sarcopenia were 4.3 years older, and their BMI was 5.7 kg/m^2^ lower when compared to the participants without sarcopenia. No significant differences were observed between the groups in terms of age (*p* = 0.094) or height (*p* = 0.520). The median weight and BMI in the sarcopenic group were 56.5 kg (IQR: 48.0–63.5) and 22.7 kg/m² (IQR: 19.9–24.5), respectively, whereas the corresponding values in the non-sarcopenic group were 72.0 kg (IQR: 64.5–80.9) and 30.4 kg/m² (IQR: 23.0–32.5), with both differences reaching statistical significance (*p* < 0.001). The gender was almost equally distributed (50.5% vs. 49.5%), whereas an unequal distribution (75% vs. 25%) was observed for the presence of comorbidities. The prevalence of comorbidities was greater among participants with sarcopenia, with 83.3% having at least one comorbidity and 95.8% having two or more (*p* < 0.001).

**Table 1:** Demographic characteristics (*n* = 96)

Variables	No Sarcopenia (*n* = 13) Median (IQR)	Sarcopenia (*n* = 83) Median (IQR)	Total (*n* = 96) Median (IQR)	*p* value*
Age in years	68.0 (65–72)	73 (65–78)	73 (65–78)	0.094
Height in cm	159.0 (148.0–173.0)	155.0 (150.0–164.5)	155.0 (155.0–165.0)	0.520
Weight in Kg	72.0 (64.5–80.9)	56.5 (48–63.5)	58.05 (48.2–66.6)	< 0.001
BMI in kg/m^2^	30.4 (23.0–32.5)	22.7 (19.9–24.5)	23.0 (20.3–25.6)	< 0.001
	Number (Percentage)			
Gender (n%)				
Male	6 (46.15%)	42 (50.60%)	48 (50.00%)	< 0.001
Female	7 (53.84%)	41 (49.40%)	48 (50.00%)	< 0.001
Comorbidities				
1 comorbidity	12 (16.66%)	60 (83.33%)	72 (75%)	< 0.001
≥ 2 comorbidities	1 (4.16%)	23 (95.83%)	24 (25%)	< 0.001

*between non-sarcopenia and sarcopenia groups

The distribution of participants in various categories of sarcopenia is depicted in Table 2. More than 46% of the participants (*n* = 45) were in the sarcopenic category, followed by 39% (*n* = 38) in the severe sarcopenia stage. Because the numbers in the possible (*n* = 6) and no sarcopenia (*n* = 7) categories were small, they were combined into a non-sarcopenic group (*n* = 13) for all analyses.

**Table 2:** Distribution of older adults in different categories of sarcopenia (*n* = 83)

Variables	Frequency *n* (%)
No sarcopenia	7 (7.3%)
Possible sarcopenia	6 (6.3%)
Sarcopenia	45 (46.9%)
Severe sarcopenia	38 (39.6%)

Table 3 presents the comparison of muscle metrics and manual dexterity parameters between older adults with and without sarcopenia. Participants with sarcopenia demonstrated significantly lower handgrip strength (median 14.9 kg, IQR: 9.1–19.0) compared to those without sarcopenia (median 19.5 kg, IQR: 18.0–27.3; *p* = 0.003, 95% CI: 2.90–11.90). Similarly, appendicular skeletal muscle mass was markedly reduced among the sarcopenic group (median 5.0 kg/m², IQR: 4.4–5.8) relative to the non-sarcopenic group (median 6.2 kg/m², IQR: 5.9–7.7; *p* = 0.007, 95% CI: 0.30–1.93). Functional performance, as measured by the five-times chair stand test, was poorer among participants with sarcopenia (median 13.1 s, IQR: 10.2–17.6) compared to their counterparts (median 10.3 s, IQR: 9.4–11.8; *p* = 0.015, 95% CI: − 5.16 to − 0.45). The strength, body composition and physical performance were 34.09%, 17.96%, and 29.14% lower among older adults who are sarcopenic when compared to non-sarcopenic.

**Table 3:** Strength, body Composition, physical performance and manual dexterity profile (*n* = 96)

Variables	No sarcopenia (*n* = 13) Median (IQR)	Sarcopenia (*n* = 83) Median (IQR)	Total (*n* = 96) Median (IQR)	*p* value* 95% CI
Hand grip strength (in kg)	19.5 (18.0–27.3)	14.9 (9.1–19.0)	15.6 (9.4–19.8)	0.003 (2.90–11.90)
Appendicular skeletal muscle mass (in kg/m^2^)	6.2 (5.9–7.7)	5.0 (4.4–5.8)	5.2 (4.5–6.1)	0.007 (0.30–1.93)
5 times chair stand test (in seconds)	10.3 (9.4–11.8)	13.1 (10.2–17.6)	12.8 (10.0–16.7.0.7)	0.015 (−0.015-−0.45)
PPT dominant side (Numbers in 30 s	11.6 (10.6–13.0)	10.3 (8.6–12.3)	10.6 (8.6–12.3)	0.043 (2.32–2.67)
PPT non-dominant side (Numbers in 30 s)	11.0 (9.6–11.6)	9.0 (7.3–10.6)	9.3 (7.3–11.0)	0.023 (0.33–3.00)
PPT both hands (Numbers in 30 s)	17.3 (16.0–18.0)	14.0 (10.0–16.5.0.5)	14.0 (10.3–17.3)	0.004 (1.33–6.00)
PPT assembly (Numbers in 60 s)	17.0 (14.0–19.0)	13.0 (8.0–17.0)	14.0 (9.0–17.2)	0.016 (1.00–9.00)

*PPT* Purdue Pegboard Test, *CI* Confidence Interval, *IQR* Interquartile range

*between non-sarcopenia and sarcopenia groups

With respect to manual dexterity, the Purdue Pegboard Test (PPT) scores were significantly lower among the sarcopenic group across all four subtasks. On the dominant hand, participants with sarcopenia completed fewer pegs (median 10.3, IQR: 8.6–12.3) than those without sarcopenia (median 11.6, IQR: 10.6–13.0; *p* = 0.043, 95% CI: 2.32–2.67). Similar differences were observed for the non-dominant hand (median 9.0 vs. 11.0; *p* = 0.023, 95% CI: 0.33–3.00), both hands combined (median 14.0 vs. 17.3; *p* = 0.004, 95% CI: 1.33–6.00), and the assembly task (median 13.0 vs. 17.0; *p* = 0.016, 95% CI: 1.00–9.00). Collectively, these findings indicate that sarcopenia is associated with reduced muscle strength and functional performance, as well as measurable declines in fine motor dexterity among older adults.

Table 4 compares manual dexterity between sarcopenic and non-sarcopenic older adults (*n* = 96). The Kruskal-Wallis test was performed since there was an unequal distribution of participants. The mean difference in scores between the two groups showed a gradual increase in the time taken to complete the task from the dominant side to assembly, ranging from 1.34 s to 4.00 s, and all were statistically significant. The 95% CI further supports the reliability of these findings.

**Table 4:** Comparison of dexterity between sarcopenic and non-sarcopenic older adults (*n* = 96)

Variables	No Sarcopenia (*n* = 13)	Sarcopenia (*n* = 83)	Mean difference	*p*-value	95% CI
PPT dominant side (Numbers in 30 s)	11.66	10.35	1.34	0.043	(2.32, 2.67)
PPT non-dominant side (Numbers in 30 s)	11.00	9.00	1.66	0.023	(0.330, 3.00)
PPT both hands (Numbers in 30 s)	17.33	14.00	3.34	0.004	(1.33, 6.00)
PPT assembly (Numbers in 60 s)	17.00	13.00	4.00	0.016	(1.00, 9.00)

*PPT* Purdue Pegboard Test, *CI* Confidence Interval

Additionally, multiple regression analysis was carried out to determine the association between MD and the components of sarcopenia. Table 5 presents the findings of the unadjusted and adjusted analyses examining the association between sarcopenia parameters and motor dexterity (MD). In the unadjusted analysis, handgrip strength (*r* = 0.34, *p* < 0.001) and the 5-times chair stand test (*r* = 0.50, *p* < 0.001) demonstrated significant correlations with MD, whereas appendicular skeletal muscle mass (ASM) did not show a significant association (*r* = 0.05, *p* = 0.630). In the multivariate model adjusted for age, gender, BMI, comorbidities, and cognitive status, handgrip strength (*r* = 0.77, *p* = 0.013, 95% CI: 0.16–1.43) and the 5-times chair stand test (*r* = 0.77, *p* = 0.03, 95% CI: − 1.24 to − 0.04) remained independently associated with MD. In contrast, the association between ASM and MD remained non-significant (*r* = 0.75, *p* = 0.73, 95% CI: − 6.32 to 4.02). These findings suggest that muscle strength and functional performance, rather than muscle mass alone, could be the principal determinants of manual dexterity among older adults.

**Table 5:** Association of manual dexterity with strength, skeletal muscle mass and physical performance (*n* = 96)

	Unadjusted analysis			Adjusted analysis*		
	*r*	*p*	95%CI	*r*	*p*	95%CI
Hand grip strength (in kg)	0.34	< 0.001	0.32–1.16	0.77	0.013	0.16–1.43
Appendicular skeletal muscle mass (in kg/m^2^)	0.05	0.630	−1.99-3.27	0.75	0.73	−6.32-4.02
5 times chair stand test (in seconds)	0.50	< 0.001	−1.67- −0.79	0.77	0.03	−1.24- −0.04

*adjusted for age, gender, BMI, comorbidities and cognitive status

## Discussion

This study aimed to compare manual dexterity between non-sarcopenic and sarcopenic older adults residing in LTCS. The result of this study showed a statistically significant decline in dexterity among sarcopenic older adults when compared to non-sarcopenic older adults.

This study had 83 sarcopenic older adults, whereas the non-sarcopenic residents of LTCS were only 13. This huge difference of 86% sarcopenic vs. 14% non-sarcopenic older adults shows the high prevalence of sarcopenia among residents of LTCS [6]. Along with age, a sub-optimal diet[22] and reduced physical activity[9] among residents of LTCS could be factors responsible for this high percentage. Older adults who were non-sarcopenic were 4.3 years younger than sarcopenic, emphasizing lower age as a protective factor [10]. This study did not show the influence of sex on sarcopenia, which follows previous literature wherein conclusive evidence of sex differences was not reported [6].

Age is reported to affect manual dexterity as older adults have poor eye-hand coordination, precision abilities, in-hand manipulation skills, and force modulation capacities [13]. These age-related declines in MD are due to the physiological changes in the neuromusculoskeletal and somatosensory systems. This study found that the presence of sarcopenia has an added effect on MD, as there was an average of 23% decline in MD evauated using PPT. The prerequisites for all the sub-tasks in PPT are good eye-hand coordination, in-hand manipulation abilities, bimanual control, and precision capacities. These activities are dependent on muscle strength and muscle mass. Among older adults, a significant decline in handgrip and finger-pinch strength, the ability to control submaximal pinch forces, and to maintain a steady precision finger pinch posture has been documented [13]. Also, a significant reduction in speed while placing small objects accurately with a finger grip is noticed; this correlates well with the scores of the PPT, as all the subtasks took more time to complete [11]. Compared to older adults without sarcopenia, those with sarcopenia had a significant decline in MD, hence indicating the role of sarcopenia in influencing MD. These results suggest that sarcopenia is not limited to gross motor weakness but may also extend to fine motor control, possibly through mechanisms such as motor unit remodelling, decreased motor neuron firing rates, and impaired sensory-motor integration [18]. The decline in dexterity may therefore represent a peripheral manifestation of broader neuromuscular degeneration associated with sarcopenia.

Most of the research on sarcopenia has studied its influence on the larger muscles, and the smaller ones are overlooked. This study documents the role of sarcopenia on the small intrinsic muscles of the hand, as those are responsible for dexterity activities [17]. More than 20 muscles, which can be broadly categorized as intrinsic hand muscles and extrinsic hand muscles, work together to precisely control hand and finger movements. Fine motor control is the general function of the intrinsic hand muscles [29], whereas gross motion performance and the generation of significant hand forces are the functions of the extrinsic hand muscles [16]. Upper extremity muscle strength is impacted by aging, with intrinsic muscles seeing a stronger reduction in strength than extrinsic muscles. This selective weakness of intrinsic muscle could be the reason for the decline in dexterity among sarcopenic older adults when compared to the non-sarcopenic group [28].

In the present study, it was observed that handgrip strength and performance on the five-times chair stand test were significantly associated with motor dexterity (MD) among older adults, even after adjusting for age, gender, body mass index, comorbidities, and cognitive status. This finding underscores the critical role of neuromuscular strength and functional performance in the maintenance of fine motor control [12]. The persistence of these associations following adjustment indicates that the relationship between sarcopenia-related parameters and MD extends beyond general age-related or systemic influences. In contrast, appendicular skeletal muscle mass did not show a significant association with MD, suggesting that muscle quantity alone may not adequately reflect the neuromuscular attributes required for precision and coordination. These observations are consistent with prior evidence demonstrating that muscle strength and physical performance are more robust predictors of functional capacity and independence than muscle mass alone [6]. The results collectively emphasize the functional dimension of sarcopenia and highlight the importance of muscle quality and neural activation efficiency in determining dexterity outcomes.

From a mechanistic standpoint, the link between reduced muscle strength and impaired dexterity may be explained by age-related motor unit remodelling, reduced motor neuron firing rates, and diminished cortical excitability, all of which compromise fine motor control [4]. These neuromuscular alterations likely contribute to delayed motor unit recruitment and reduced synchronization during precision tasks. Furthermore, lower limb function, as captured by the chair stand test, may indirectly influence upper limb dexterity through shared pathways governing balance, coordination, and global motor control [24]. Clinically, these findings suggest that interventions emphasizing progressive resistance training, task-specific motor practice, and neuromuscular re-education may offer superior benefits for preserving fine motor skills in older adults, compared with strategies focused solely on maintaining muscle mass. Future longitudinal and interventional studies are warranted to clarify causal pathways and to determine whether strengthening and coordination-based programs can mitigate the decline in dexterity associated with sarcopenia.

To our knowledge, studies on manual dexterity among older adults residing in LTCS in India are limited. It carries significance as it studies the influence of sarcopenia on manual dexterity. This study is not devoid of a few limitations. Firstly, there was an uneven distribution of participants in the groups. Secondly, sarcopenia and MD evaluations were carried out by the same investigator; hence, an evaluator bias cannot be ruled out. Thirdly, an assessment of the participants’ nutritional status, amount of assistance needed, or physical activity level was not done.

## Clinical significance

This study links the impact of physiological decline due to sarcopenia to MD, a prerequisite for functional activities and independence. Integrating assessments and rehabilitation for sarcopenia and consequent decline in MD can help mitigate these adverse effects. Also, rehabilitation professionals should incorporate dexterity-based activity programs to maintain functional independence and thereby QoL.

## Conclusion

The influence of sarcopenia on manual dexterity among older adults residing in the LTCS has been identified in this study. There is a decline of 12% to 33.9% in various subsets of manual dexterity among sarcopenic older adults when compared to non-sarcopenic older adults residing in LTCS.

## Data Availability

The data that support the findings of this study are available from the corresponding author, [Girish N], upon reasonable request.
